# let-7i-5p, miR-181a-2-3p and EGF/PI3K/SOX2 axis coordinate to maintain cancer stem cell population in cervical cancer

**DOI:** 10.1038/s41598-018-26292-w

**Published:** 2018-05-18

**Authors:** Ravindresh Chhabra

**Affiliations:** Department of Biotechnology, Panjab University, Chandigarh, 160014 India

## Abstract

The characteristics of cancer stem cells (CSCs) and the genes responsible for their maintenance are highly variable in different cancers. Here, we identify the coordination among miRNAs and EGF pathway genes which is critical for the maintenance of CSCs in cervical cancer. The transcript analysis of CSCs enriched from cervical cancer cell lines (CaSki and HeLa) revealed a significant upregulation of SOX2. Since EGF receptor is frequently over expressed in cervical cancer, we hypothesized that EGF pathway may be responsible for the upregulation of SOX2. Also, the media used for CSC enrichment was supplemented with EGF. The hypothesis was validated as inhibiting the EGF/PI3K pathway suppressed the expression of SOX2 and reduced the CSC population. In addition, miRNA profiling identified miR-181a-2-3p and let-7i-5p as markedly reduced in CSCs. The exogenous expression of either of these miRNAs in CaSki cells inhibited the expression of SOX2 and subsequently reduced CSC population. In conclusion, this study highlights for the first time the contrasting role of let-7i-5p/ miR-181a-2-3p and EGF/PI3K/SOX2 axis in maintaining cervical CSCs. While the EGF pathway promotes CSC formation in cervical cancer by inducing SOX2, miR-181a-2-3p/let-7i-5p counteracts the EGF pathway by inhibiting SOX2, thereby reducing the CSC population.

## Introduction

Cervical cancer is among the leading causes of mortality in women^1^. Although in the recent years, there has been a remarkable decrease in the number of deaths associated with this disease owing to the enhanced awareness, early diagnosis and the availability of effective vaccines including gardasil and cervarix in the market^2^. However the fatalities of cervical cancer continue unabated in developing countries including India because of the socioeconomic reasons and low adoption rate of vaccines^1^. Many a times, the cervical cancer is detected at a later stage where the existing therapies against the disease are rendered ineffective and even if they work, there is a greater chance of relapse of the disease^2^. Hence, there is an imminent need to look for novel and effective ways of countering the disease. In the past decade, the cancer stem cells (CSCs) have been the subject of intensive research. They were initially discovered in leukemia and lymphomas^3^ but have eventually been shown to exist in almost all types of solid tumors including breast^4^, brain^5,6^, colon^7,8^ and pancreas^9^. The CSCs signify a novel paradigm in cancer biology as they have been implicated in origin of cancer^10–12^, chemoresistance^13^, radioresistance^14^ and metastasis^15,16^. The higher proportion of CSCs in a tumor has often been associated with more aggressive tumors and reduced survival rate in cancer patients^17–20^.

Bortolomai *et al*. were the first to report CSCs in cervical cancer in 2010^21^. They used spheroid method to isolate CSCs from cervical cancer cell lines and showed that these CSCs were highly tumorogenic and had marked increase in radioresistance. Surprisingly, these cells have significantly higher expression of CD49f but had no change in CD44 (CSC marker in breast^4^, colon^22^, ovarian^23^, prostate^24^ and pancreatic cells^9^), thereby implying the variability in the CSCs of different cancers. Since then, the cervical CSCs have been isolated using SOX2^25^, BCRP1^26^ in addition to the use of generic CSC markers including ALDH^27^ and sidepopulation assay^28,29^. The cervical CSCs isolated by varied methods were highly tumorogenic^21,26–29^, invasive^26^, and had enhanced rate of self-renewal^27^, radioresistance^21,28^ and chemoresistance^27,28^. CD49f (ITGA6), a cell surface marker protein was found to be enhanced in cervical CSCs^25,30^ and hence could be considered as an alternative cervical CSC marker. There are however contrasting reports on another cell surface marker, CD133 in cervical CSCs which was observed to be significantly upregulated in one study^28^ but not in the other^30^. Recently, miR-145^31^ and miR-23b^32^ have been shown to regulate cervical CSCs. Both miR-145 and miR-23b were found to be underexpressed in cervical CSCs and their exogenous expression suppressed the CSC population^31,32^. This was attributed to the reduction of SOX2, OCT4 and NANOG by miR-145^31^ and the reduction of ALDH1A by miR-23b^32^.

The existing research on cervical CSCs is inadequate and none of the previous studies details the pathways responsible for regulating cervical CSCs. Therefore, this study was designed to identify the key genes, miRNAs and underlying pathways critical for the maintenance of CSCs in cervical cancer.

## Materials and Methods

### Cell Culture and transfections

CaSki and HeLa cells were obtained from National Centre for Cell Science, Pune, India and maintained in DMEM medium containing 10% (v/v) fetal calf serum and 1 × antibiotic-antimycotic solution (HiMedia) in a humidified 5% CO_2_ atmosphere.

For over expression studies, the cells were transfected in 6-well plates by using lipofectamine 2000 with either 3 μg of the miRNA clones/shSOX2 clone. The cells were harvested 24 h or 48 h post transfection, as indicated.

### ALDH assay

ALDH activity was analyzed using PicoProbe Aldehyde Dehydrogenase Activity Fluorometric Assay kit (BioVision Inc., Milpitas, CA) as per the manufacturer’s instructions. Briefly, in this assay, acetaldehyde is oxidized by ALDH to generate NADH which couples with the probe to generate a potent fluorescence. CaSki cells or CSCs derived from CaSki cells (1 × 10^6^) were lysed by 200 μl ALDH Assay Buffer followed by centrifugation at 12,000 rpm for 5 min and the supernatant was collected. 10 μl of the cell lysate was used in reaction mix containing the substrate for the assay. Fluorescence was quantified at 535 nm at 10 min and 30 min intervals. The experiment was repeated three times and all tests were done in duplicates.

### Pharmacological inhibitor treatment

EGFR phosphorylation inhibitor, Erlotinib Hydrochloride and PI3K inhibitor, LY294002 were purchased from Sigma Aldrich. The cells were serum starved for 12 h and then treated with either 1 μM Erlotinib Hydrochloride or 15 μM LY294002 for 48 h, as indicated. The medium (containing fresh inhibitors) was replaced after 24 h. The cells treated with DMSO were used as the reference set. Following the treatment, the cells were harvested for real time PCR, sphere formation and clonogenic assay.

### shRNA construction and miRNA clones

The shRNA oligo sequences against SOX2 were taken from the existing literature (Chew *et al*. 2005) and modified according to our desired restriction sites Hind III and BamH1 for cloning in pEGFP-N3 vector. The oligos synthesized were 5′-*AGCTT*CC**GAAGGAGCACCCGGATTA**TTTCAAGAGAATAATCCGGGTGCTCCTTCTTTTT*G*-3′, 5′-*GATCC*AAAAA**GAAGGAGCACCCGGATTA**TTCTCTTGAAATAATCCGGGTGCTCCTTCGG*A*-3′; the sequence in italics show restriction sites and the sequence in bold shows target sequence against SOX2. The oligos were annealed by keeping them at 95 °C and gradually decreasing the temperature by 1 °C per minute till it reaches 25 °C. The annealed oligos were ligated with pEGFP-N3 vector digested with Hind III and BamH1 and transformed in *E coli* DH5α. The plasmid was isolated from the transformed cells and sequenced to confirm the presence of shRNA oligos in the plasmid. The resulting plasmid was referred to as shSOX2.

miRNA expression plasmids for the exogenous expression of miR-181a-2-3p (SC400203) and let-7i-5p (SC400011) were purchased from OriGene Technologies, Inc. In these expression plasmids, the miRNA precursors are cloned into pCMV-MIR vector via SgfI and MluI site.

The endotoxin free plasmids for transfection studies were prepared by the ZymoPURE Plasmid Maxiprep Kit (Zymo Research, USA).

### Sphere formation assay

Single cell suspension of HeLa and CaSki cell lines (1200 cells per well) was plated in 24 well ultralow attachment plate (Corning Inc., USA). These cells were cultivated for 7 days in serum free DMEM medium supplemented with 20 ng/ml EGF and 20 ng/ml bFGF and 1 ml of 50 × B27 under normal conditions. The spheres were counted manually under inverted phase contrast microscope. All the experiments were repeated three times.

### Clonogenic assay

Single cell suspension of CaSki cells were plated at a density of 2000 cells per well in 6 well plate and cultured for 10 days in DMEM medium containing 10% (v/v) fetal calf serum and 1 × antibiotic-antimycotic solution. The media was replaced every 48 h. The colonies were fixed using 95% ethanol for 30 minutes followed by staining with 0.5% crystal violet prepared in 2% ethanol for 15 minutes. The extra stain was washed with distilled water and the pictures of stained colonies were taken.

For quantitative analysis, the stained colonies were dissolved in 30% glacial acetic acid and the absorbance was taken at 570 nm using plate reader.

### Small RNA sequencing

The RNA samples were outsourced for quality testing, small RNA sequencing and bioinformatics analysis to Scigenom labs, Cochin, Kerala (India). In brief, total RNA was extracted using Trizol reagent (Invitrogen, CA, USA) and the quality was analyzed on Agilent Technologies Tapestation. The samples with RNA Integrity Number (RIN) greater than or equal to 8 were used for small RNA library preparation by Illumina TruSeq small RNA sample preparation kit as per the manufacturer’s instructions. The libraries were then sequenced on Illumina HiSeq. 2500 with a 1 × 50 bp reads and the data was processed to generate FASTQ files. The adapter sequences and non-coding RNA other than miRNAs were removed. The unique reads with length 17–35 bp were aligned to miRBase-21 mature and precursor sequences of *Homo Sapiens* using Bowtie program (version 0.12.9) to identify known miRNAs obtained in the experiment. The differential expression analysis of miRNAs between CaSKi cells and CSCs derived from CaSki cells was determined by counting the aligned reads using the statistics package DESeq available in R for differential expression studies. The package works based on binomial distribution and estimates the distribution variants between the sample reads.

### Real time PCR

Total RNA was extracted using Trizol reagent (Invitrogen, CA, USA) and reverse transcription was carried out with random hexamer or miRNA specific primer using Verso cDNA synthesis kit (Thermo Scientific, MA, USA) according to the manufacturer’s protocol. Real time PCR was done using SYBR Green PCR master mix (Applied Biosystems, CA, USA). Results were normalized with 18 s rRNA and the data was analyzed using Pfaffl’s method^33^. For the miRNA quantification, stem loop primers were synthesized. The primers are listed in Supplementary Tables 1 and 2.

### Protein preparation and western blot analysis

The cells were trypsinized and cell pellets were lysed with modified RIPA buffer (50 mM Tris-HCl, pH 7.4, 150 mM NaCl, 1 mM EDTA, 1% NP40, 0.25% Na deoxycholate, 1 × protease inhibitor cocktail and 1 mM PMSF) and kept in ice for 30 min with intermittent vortexing. Lysates were centrifuged at 12,000 rpm for 30 min and supernatant was collected. Protein concentration was determined using the BCA reagent. Equal amounts of protein (~50 μg) were separated by 12% sodium dodecyl sulphate-polyacrylamide gel electrophoresis (SDS-PAGE) and transferred to PVDF membrane (Mdi, Advanced Microdevices, India). The membrane was blocked with 3% skim milk in Tris buffered saline (20 mM Tris, 150 mM NaCl, pH 7.4) with 0.1% Tween-20 for 1 h and then incubated with primary antibody (AKT1/2/3, GAPDH, HMGA2, phosphorylated AKT1/2/3, PI3kinase p55γ, SOX2) in 1% skim milk for 3 h followed by incubation with appropriate secondary antibody (anti-mouse/anti-rabbit HRP linked) for 1 h. Blots were developed using the enhanced chemilumiscence ECL western blot detection system (Pierce). Equal loading of protein was confirmed using GAPDH antibody. The experiment was repeated at least three times and representative results are presented. The quantitative analysis of western blotting was done by estimating the signal intensity using AlphaImager 3400 (Alpha InnoTech Corporation, CA, USA). The integrated density values (IDV) value was calculated as the density values of the specific protein bands/GAPDH density values. The fold change was calculated by dividing this value for transfected cells by the value for control samples. The experiments were repeated at least three times and the representative results are shown.

### Statistical analysis

All the experiments were repeated a minimum of 3 times and data are presented as mean ± standard deviation (SD). Statistical significance, wherever indicated was calculated using t-test. A probability value of less than 0.05 was considered to be statistically significant.

## Results

### Enrichment of cancer stem cells (CSCs) from cervical cancer cell lines

**Figure 1:**
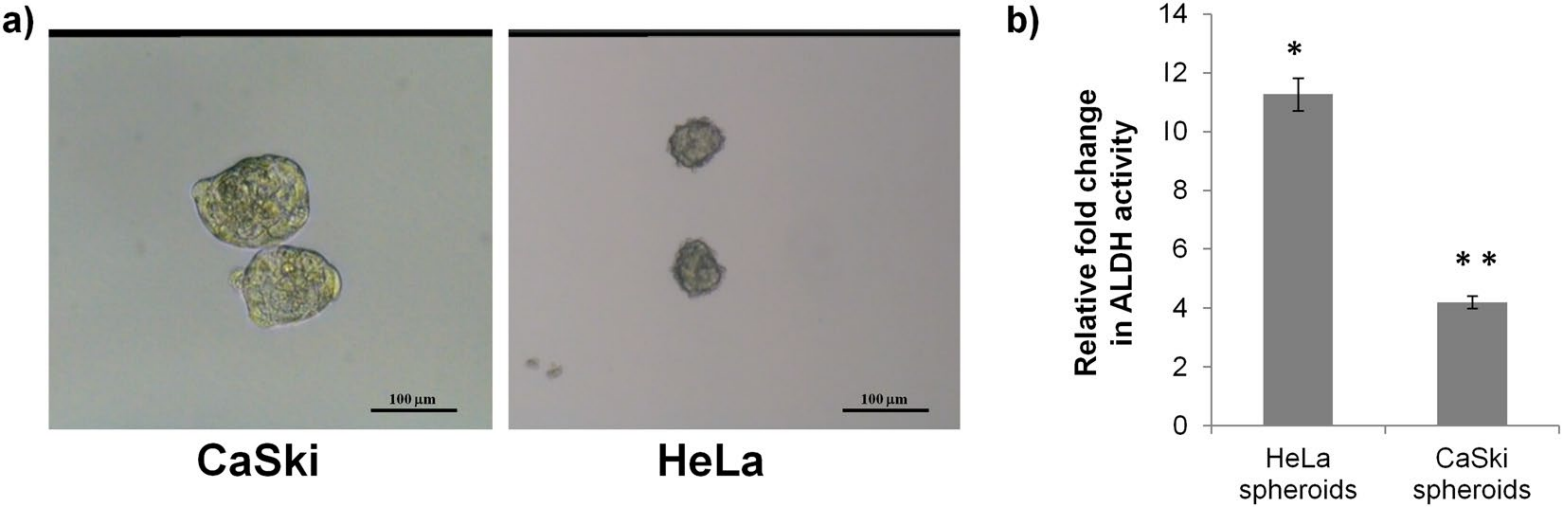
Enrichment of cancer stem cells (CSCs) from cervical cancer cell lines. (**a**) HeLa and CaSki cells were cultured on ultra-low attachment plates in serum free DMEM media supplemented with EGF, FGF and B27 complement and allowed to grow for 7 days. This method allows the growth of stem-like cells as spheroids while the non-stem cells undergo anoikis. The images have been taken at 100X magnification. (**b**) Aldehyde dehydrogenase (ALDH) assay revealed that the ALDH activity in the HeLa and CaSki spheroids increased by 11- and 4-fold, respectively, thereby confirming the enrichment of CSCs in both the cell lines by spheroid formation method. *p value < 0.05, **p value < 0.01.

The CSC population from CaSki and HeLa cells was enriched using spheroid culture method. The cells were plated at extremely low density (12,000 cells per well) on 6-well ultra low attachment plates for 7 days in serum free media supplemented with 20 ng/ml EGF and 20 ng/ml bFGF and 1 × B27. These conditions allow the stem-like cells to proliferate and form spheres while the non-stem population undergo anoikis. Figure 1a shows a representative image of the spheres formed from both the cell lines. Since enhanced aldehyde dehydrogenase (ALDH) activity is one of the hallmarks of the cancer stem cell phenotype, the stemness characteristics of the isolated spheroids was further confirmed by ALDH activity assay. There was about 4-fold increase in ALDH activity in CaSki spheroids and 11-fold increase in HeLa spheroids when compared with their respective cell types (Fig. 1b).

### SOX2 is essential for maintaining CSC subpopulation in cervical cancer cell lines

**Figure 2:**
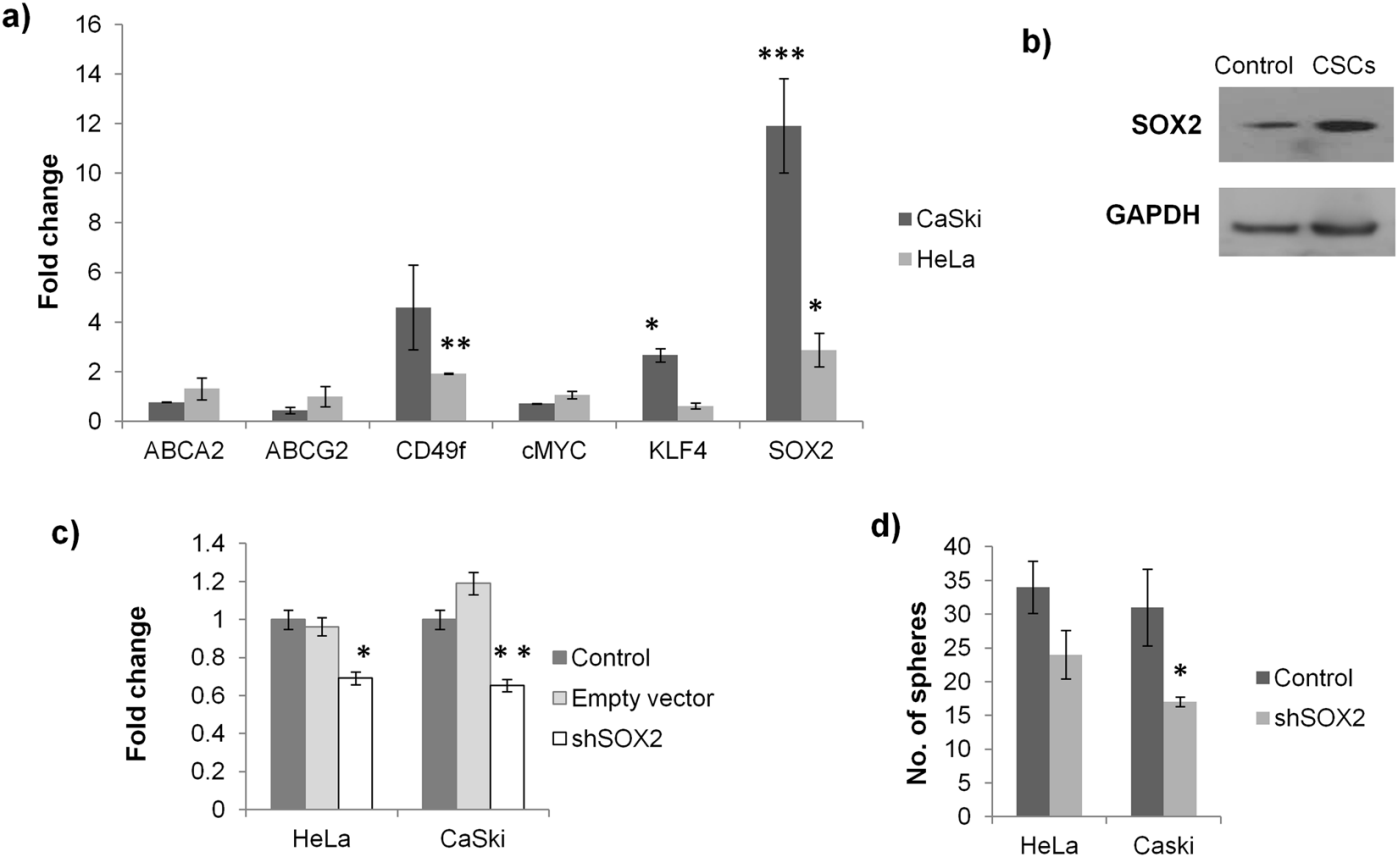
SOX2 silencing suppresses CSC phenotype. (**a**) Real time PCR for stem cell marker genes revealed 4.59, 2.67 and 11.91-fold change in CD49f, KLF4 and SOX2, respectively in CaSki CSCs when compared with CaSki cells and 1.92, 0.61 and 2.87-fold change in CD49f, KLF4 and SOX2, respectively in HeLa CSCs when compared with HeLa cells. No significant change was observed in ABCA2, ABCG2 and cMYC. (**b**) The increased expression of SOX2 was also confirmed at the protein level in CaSki spheroids by western blotting. (**c**) The forced expression of shSOX2 reduced the SOX2 expression by 31% and 35% in HeLa and CaSki cells, respectively in comparison to the untransfected sample. Empty vector implies the pEGFP-N3 without any insert and control implies the untransfected cells. (**d**) The number of spheres formed by the cells transfected with shSOX2 decreased by 30% and 45% in HeLa and CaSki cells, respectively. *p value < 0.05, **p value < 0.01, ***p value < 0.001.

In order to identify the genes responsible for maintenance of CSCs, the transcript analysis of stem cell marker genes (ABCA2, ABCG2, cMYC, CD49f, KLF4 and SOX2) was carried out by real time PCR. Out of these, there was significant upregulation of CD49f (4.5-fold), KLF4 (2.8-fold) and SOX2 (10.4-fold) in CaSki CSCs while there was no significant change in the other transcripts (Fig. 2a). CD49f and SOX2 have previously been described to be enhanced in cervical CSCs^21,25,30^, thereby supporting our results. The real-time PCR analysis for HeLa CSCs also showed upregulation of SOX2 (2.9-fold) and CD49f (1.9-fold) but the level of KLF4 (0.61-fold) was surprisingly found to be reduced. This evidently indicates that the stemness in different cell lines may be maintained by coordination of different stem-cell genes. However, in both the cervical cancer cell lines, HeLa and CaSki, the expression of SOX2 was significantly upregulated. The increased SOX2 expression was also observed at the protein level in CaSki CSCs (Fig. 2b). To ascertain the importance of SOX2 for maintaining the CSCs, SOX2 was silenced using shSOX2 and sphere formation assay was used to determine its effect on CSCs. The SOX2 silencing reduced the SOX2 levels to 0.69- and 0.65- fold in HeLa and CaSki cells, respectively (Fig. 2c) and decreased the number of spheres formed from 34 to 24 in HeLa and from 31 to 17 in CaSki cells (Fig. 2d).

### EGF/PI3K pathway modulates the CSC phenotype

**Figure 3:**
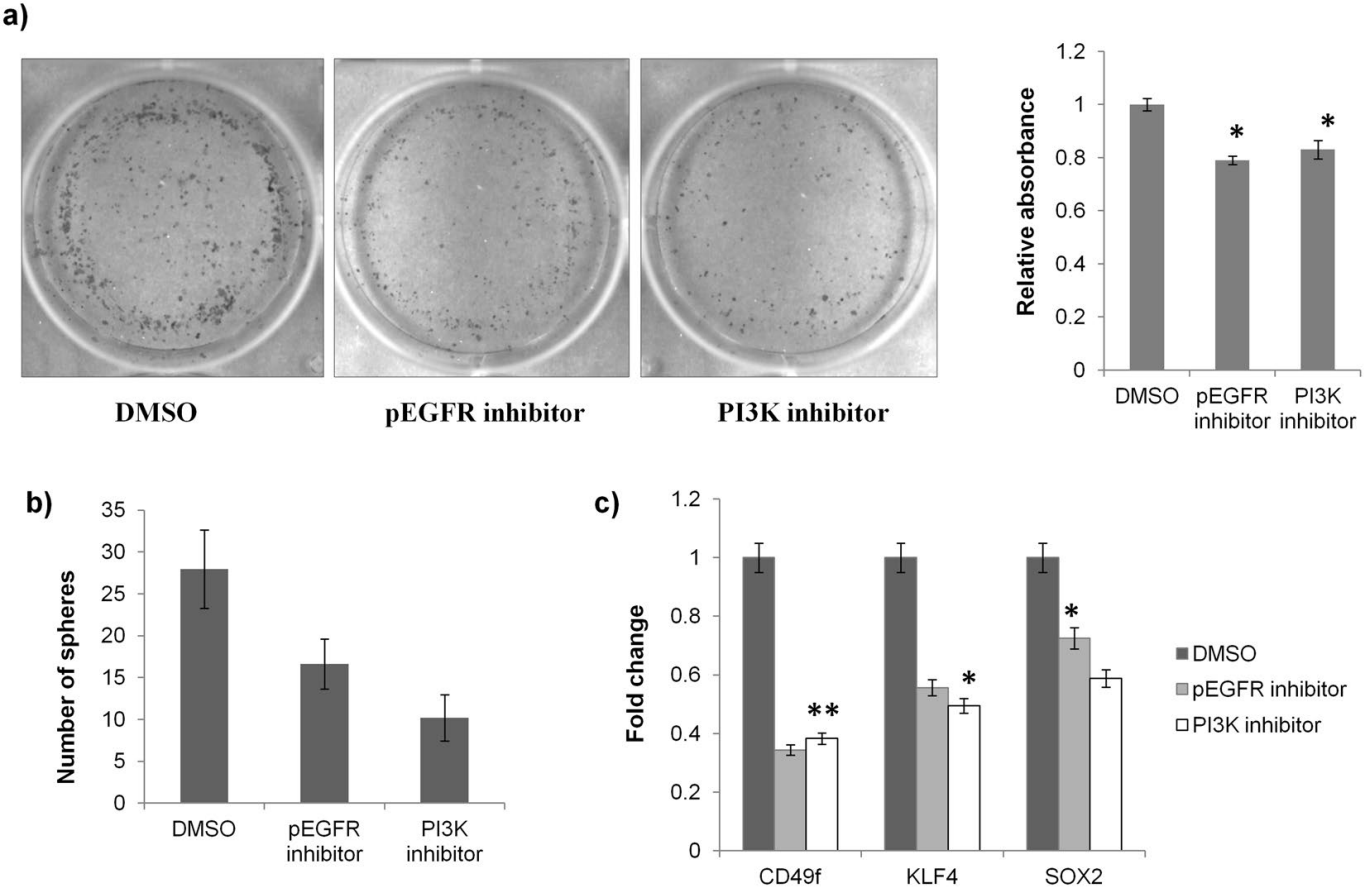
EGF/PI3K/SOX2 axis is responsible for CSC maintenance in CaSKi cells. (**a**) Clonogenic assay: CaSki cells treated with pEGFR inhibitor or PI3K inhibitor for 48 h were harvested and 2000 cells were plated in one well of 6-well plate and allowed to grow for a period of 10 days. The cells were then stained with crystal violet. The number of colonies reduced significantly in cells treated with either pEGFR inhibitor or PI3K inhibitor when compared with DMSO treated cells. The quantitative change was calculated by dissolving the stained colonies in 30% glacial acetic acid and recording their absorbance at 570 nm. The decrease was about 21% and 17% in cells treated with pEGFR inhibitor and PI3K inhibitor, respectively. (**b**) Sphere formation assay: CaSki cells were treated with pEGFR inhibitor or PI3K inhibitor for 48 h and then assayed for sphere formation. The number of spheres reduced from 28 in DMSO treated cells to 16 in cells treated with pEGFR inhibitor (42% decrease) and 10 in cells treated with PI3K inhibitor (64% decrease). (**c**) Real time PCR: The expression of stem cell marker genes, CD49f, KLF4 and SOX2 also reduced significantly after treatment with pEGFR inhibitor or PI3K inhibitor. *p value < 0.05, **p value < 0.01

We hypothesized that the EGF pathway may be the upstream regulator of SOX2 in cervical CSCs. This was because EGFR is frequently over expressed in cervical cancer^34,35^, thus providing the biological significance for exploring this hypothesis. Also, the CSC enrichment in this study was carried out by growing them in media supplemented with EGF, so it is likely that EGF pathway somehow induced the expression of SOX2. A study had previously shown that EGFR signalling could modulate SOX2 expression in NSCLC^36^. To validate this hypothesis, we used pharmacological inhibitors against phospho-EGFR (Erlotinib hydrochloride) and PI3K (LY294002). The pEGFR inhibitor reduced CD49f (0.34-fold), KLF4 (0.56-fold) and SOX2 (0.72-fold) and PI3K inhibitor reduced CD49f (0.34-fold), KLF4 (0.56-fold) and SOX2 (0.72-fold) in CaSki cells (Fig. 3a). The effect of pEGFR and PI3K inhibition on CSCs was also evaluated by sphere formation and clonogenic assay. The number of spheroids formed by treated CaSki cells reduced from 28 to 16 after pEGFR inhibition and from 28 to 10 after PI3K inhibition (Fig. 3b). The clonogenic assay further substantiated the effect of reduced stemness characteristics after treatment with pEGFR and PI3K inhibitors as the number of colonies reduced significantly following pEGFR and PI3K inhibition (Fig. 3c).

### Differential miRNA expression in cervical CSCs

**Figure 4:**
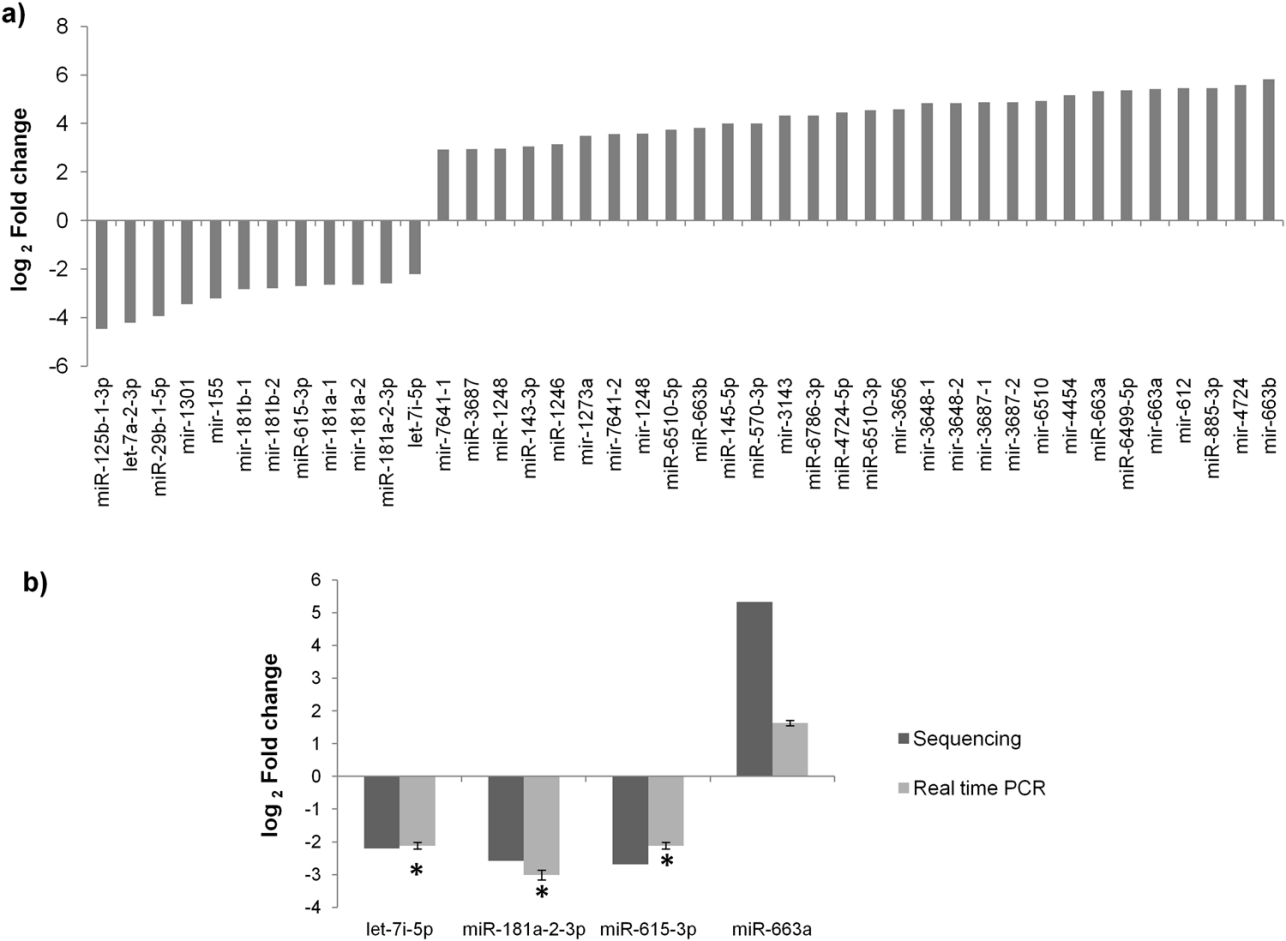
miRNA profiling in CaSki CSCs and validation of differentially expressed miRNAs. (**a**) The small RNA sequencing was carried out for CaSki cells and CSCs enriched from CaSki cell line. This experiment identified 42 differentially expressed miRNAs (12 downregulated and 30 upregulated) in CSCs with a p value < 0.05. As per the miRNA nomenclature guidelines, the mature miRNA sequences are mentioned as miR- and precursor sequences as mir-. (**b**) miRNAs (let-7i-5p, miR-181a-2-3p, miR-615-3p, and miR-663a) were selected from the list of differentially expressed miRNAs for further validation. The expression of these miRNAs were analyzed by real time PCR and found to be in concordance with the data obtained from small RNA sequencing experiment. *p value < 0.05.

The role of miRNAs in the maintenance of cervical CSCs was determined by small RNA sequencing of CaSki cells and CSCs enriched from CaSki cells. A total of 702 sequences in CSCs and 940 sequences in CaSki cells got aligned with mature miRNA sequences and 447 sequences in CSCs and 637 sequences in CaSki cells got aligned with precursor miRNA sequences in miRbase database. Among these, 629 mature and 396 precursor miRNA sequences were identified in both CaSki cells and CSCs. 42 miRNAs were found to be differentially expressed (12 downregulated and 30 upregulated) in CSCs with a p value < 0.05 (Fig. 4a).

The sequencing result was validated by performing miRNA expression analysis using real time PCR. let-7i-p, miR-181a-2-3p, miR-615-3p and miR-663a were selected for this assay. As seen in Fig. 4b, the real time data was in concordance with the sequencing data. The log_2_ fold change for let-7i-p, was −2.2 in the sequencing data and −2.1 in the real time data; for miR-181a-2-3p, log_2_ fold change was −2.6 in the sequencing data and −3.0 in the real time data; for miR-615-3p, log_2_ fold change was −2.7 in the sequencing data and −2.1 in the real time data and for miR-663a, log_2_ fold change was 5.3 in the sequencing data and 1.6 in the real time data

### Exogenous expression of let-7i-5p and miR-181a-2-3p reduces the CSC phenotype through PI3K and SOX2

**Figure 5:**
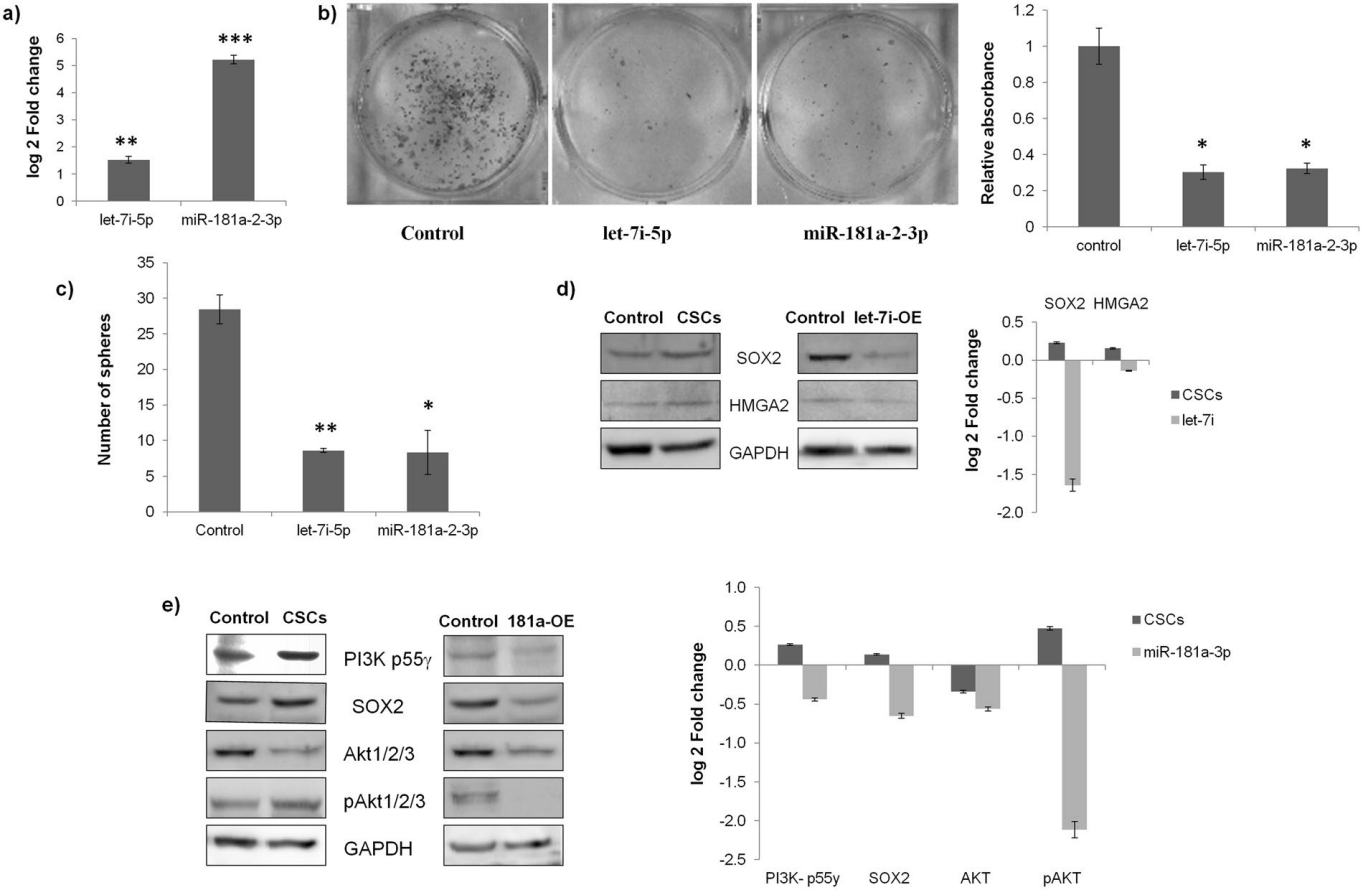
let-7i-5p and miR-181a-2-3p modulates cervical CSCs via EGF/PI3K/SOX2 pathway. (**a**) Forced expression of let-7i and miR-181a-2: The clones expressing let-7i-5p and miR-181a-2-3p were purchased from Origene and transfected by Lipofectamine 2000 in CaSki cell lines and the efficacy of the clones was examined by real time PCR. The transfection of the respective clones resulted in an increase of 1.53 log_2_ fold change in let-7i-5p and an increase of 5.21 log_2_ fold change in miR-181a-2-3p. (**b**) Clonogenic assay: The number of colonies formed by CaSki cells transfected with let-7i-5p or miR-181a-2-3p reduced significantly in comparison to the untransfected CaSki cells. The quantitative change was calculated by dissolving the stained colonies in 30% glacial acetic acid and recording their absorbance at 570 nm. The decrease was about 66.5% and 65% in let-7i-5p and miR-181a-2-3p overexpressed cells, respectively. (**c**) Sphere formation assay: The number of spheres reduced from 28 in untransfected CaSki cells to 9 in cells transfected with let-7i-5p (67% decrease) and 8 in cells transfected with miR-181a-2-3p (71% decrease). (**d**) let-7i-5p indirectly targets SOX2 through HMGA2: The western blotting data shows that the forced expression of let-7i-5p reduces the expression of HMGA2 and SOX2 to −0.14 and −1.64 log_2_ fold change, respectively. This is in significant contrast to the increase in HMGA2 and SOX2 expression in cervical CSCs. The blots were cropped according to the molecular weight of the proteins and then probed with specific antibodies. Where molecular weight of the proteins was similar, for instance SOX2 (34 kDa) and GAPDH (37 kDa), the blot was first probed with one antibody, stripped and then reprobed with another antibody. (**e**) miR-181a-2-3p targets EGF/PI3K/SOX2 pathway: The western blotting data confirms that EGF/PI3K/SOX2 pathway is activated in the CSCs of CaSki cells. The exogenous expression of miR-181a-2-3p reverses the expression of EGF pathway genes in CaSki cells. miR-181a-2-3p inhibits the expression of PI3K55γ (a validated target of miR-181a-2-3p), SOX2 and the phosphorylated AKT. The reverse pattern is clearly depicted in the densitometric analysis of the western blot data. The blots were cropped according to the molecular weight of the proteins and then probed with specific antibodies. Where molecular weight of the proteins was similar, for instance in case of SOX2 (34 kDa) and GAPDH (37 kDa) or AKT and pAKT (~55 kDa), the blot was first probed with one antibody, stripped and then reprobed with another antibody. *p value < 0.05, **p value < 0.01, ***p value < 0.001.

The exogenous expression of let-7i and miR-181a-2 in CaSki cells enhanced the expression of let-7i-5p by 5 log_2_ fold and miR-181a-2-3p by 1.5 log2 fold, respectively (Fig. 5a). The overexpression of miR-181a-2-3p and let-7i-5p in CaSki cells resulted in significant reduction in the proliferation potential of cells as indicated by the clonogenic assay (Fig. 5b). The quantitative analysis of clonogenic assay revealed that the reduction was about 60% following overexpression of either miR-181a-2-3p or let-7i-5p in CaSki cells. Both these miRNAs were also detrimental to the sphere formation as miR-181a-2-3p decreased the number of spheres by 67% and let-7i-5p reduced the number of spheres by 71% (Fig. 5c).

**Figure 6:**
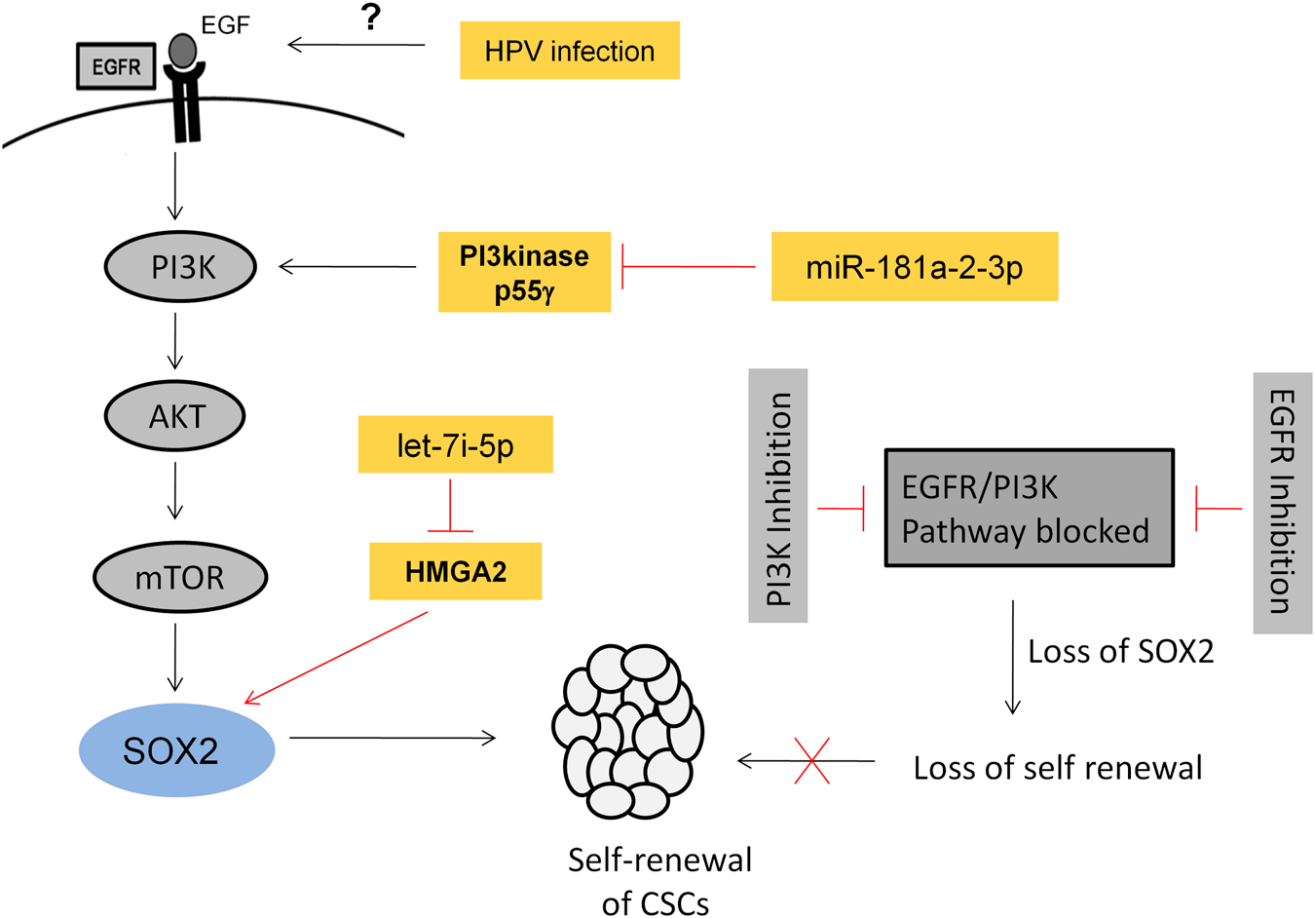
Schematic diagram explaining the mechanism of CSC maintenance in cervical cancer via let-7i-5p, miR-181a-2-3p and EGF/PI3K/SOX2 axis. In this study, it was observed that exogenous EGF added to the media used for CSC enrichment induces the expression of PI3K and phosphorylates AKT, leading to the enhanced expression of SOX2. SOX2 silencing drastically reduced the CSC population in CaSki cells. The inhibition of EGFR phosphorylation and PI3K reduced the expression of SOX2 and subsequently suppressed the CSC population, thereby establishing the role of EGF pathway in the maintenance of cervical CSCs. While EGF pathway promotes CSC maintenance, let-7i-5p and miR-181a-2-3p counteracts by indirectly targeting SOX2 and thereby suppressing the cervical CSCs. let-7i-5p inhibits HMGA2 (an inducer of SOX2 expression) and miR-181a-2-3p inhibits PI3kinase p55γ (PI3K activator). Hence, activation of EGF pathway and inhibition of let-7i-5p and miR-181a-2-3p are vital for cervical CSC maintenance. The reported literature suggests that HPV infection may cause activation of EGF pathway. If proven, this would perhaps explain the formation of cervical CSCs and hence the origin of cervical cancer.

The next objective was to determine the mechanism through which these miRNAs are able to exert this effect. Coincidentally, one of the potential pathways for both these miRNAs could be EGF/PI3K/Akt pathway (Fig. 6). This is because let-7i-5p inhibits the expression of HMGA2, an inducer of SOX2 expression^37,38^ and miR-181a-2-3p inhibits PI3kinase p55γ, PI3K activator. So the overexpression of either let-7i-5p or miR-181a-2-3p would reduce SOX2, subsequently leading to suppression of CSCs in CaSki. The western blot analysis confirms that overexpression of let-7i-5p in CaSki cells reversed the expression of HMGA2 and SOX2 in comparison to the CSCs (Fig. 5d). The expression of HMGA2, SOX2 in CSCs was 0.16, and 0.23 log_2_ fold, respectively and the expression of HMGA2, SOX2 in let-7i-5p overexpressed cells was −0.14 and −1.64 log_2_ fold, respectively.

Similarly, it was observed that the overexpression of miR-181a-2-3p reversed the expression of several proteins in EGF/PI3K/Akt pathway in comparison to the CSCs (Fig. 5e). The expression of PI3kinase p55γ, SOX2, AKT, pAKT in CSCs was 0.26, 0.14, −0.34 and 0.47 log_2_ fold, respectively and the expression of PI3kinase p55γ, SOX2, AKT, pAKT in miR-181a-2-3p overexpressed cells was −0.44, −0.65, −0.56 and −2.11 log_2_ fold, respectively. CaSki cells were considered to be the control reference for calculating the fold change. Although the expression of AKT was downregulated in both CSCs and miR-181a-2-3p overexpressed cells, the expression of the activated form of AKT (pAKT) followed the opposite trend as was seen for PI3kinase p55γ and SOX2. This proves that miR-181a-2-3p could regulate CSC maintenance through EGF/PI3K/SOX2 pathway.

## Discussion

The cancer stem cell model suggests that a small population of stem-like cells is responsible for the origin as well as progression of cancer. The conventional therapies aimed at targeting the proliferating population of the tumor would reduce the overall size of the tumor but CSCs would remain unaffected owing to their chemoresistant properties. This implies that unless CSCs are eliminated, the ideal cancer therapy will remain an elusive dream.

The cervical CSCs are relatively less studied and there is a lack of information about the pathways responsible for maintenance of cervical CSCs. Herein, the CSCs from cervical cancer cell lines were isolated by the spheroid culture method and the stemness of the CSCs was further confirmed by enhanced activity of aldehyde dehydrogenase, an enzyme which is often upregulated in stem-like cells in cancer^39,40^. The transcript analysis showed that the expression of CD49f, KLF4 and SOX2 is upregulated by 4.6-, 2.7- and 11.9-fold, respectively in CSCs. The increased expression of CD49f and SOX2 in cervical CSCs had also been reported earlier^21,25,30^. The remarkable increase in SOX2 clearly pointed to its imperative role in CSC maintenance, which was corroborated by the significant drop in CaSki CSCs following SOX2 silencing (Fig. 2). The importance of SOX2 as a stem cell marker gene is already well established. SOX2 is one of the four transcription factors used to create induced pluripotent stem cells^41^. Next, we focused on identifying the pathway responsible for enhanced expression of SOX2 in cervical CSCs. In our study, the media used for CSC isolation was supplemented with EGF and there is evidence in the literature which suggest that EGF pathway may induce SOX2^36^. Also, EGF receptor (EGFR) is frequently found to be upregulated in cervical cancer and the EGFR amplification is associated with poor prognosis and shorter patient survival^34,35^, thereby establishing the biological relevance for exploring the significance of EGF in cervical CSCs. The role of EGF pathway was confirmed as blocking the pathway by either pEGFR inhibitor or PI3K inhibitor not only reduced the expression of SOX2 but also suppressed the CSC subpopulation (Fig. 3).

The small RNA sequencing experiment identified miR-181a-2-3p and let-7i-5p as significantly downregulated in CSCs. This was remarkable as both these miRNAs have been shown to indirectly inhibit the expression of SOX2. While let-7i-5p inhibits HMGA2 (an inducer of SOX2 expression)^37,38^, miR-181a-2-3p inhibits PI3kinase p55γ (PI3K activator)^42^. The forced expression of let-7i-5p or miR-181a-2-3p in CaSki cells was shown to counteract the EGF/ PI3K pathway by indirectly targeting SOX2 and thereby suppressing the cervical CSCs (Fig. 5).

Although many possible ways have been described for the origin of cancer stem cells, it still remains a mystery^43^. Since EGF pathway seems to prompt the cervical CSC formation, the cause of EGF pathway activation could explain the origin of cervical CSCs. Cervical cancer is often associated with HPV infection and intriguingly, HPV 16 E5 gene has been observed to increase the expression of EGFR by preventing the degradation of internalized EGFR^44^. Moreover, HPV 16 E6/E7 were also shown to enhance EGFR levels^45^. Thus, one could speculate that HPV infection in cervix stimulates cancer stem cell formation via EGF pathway which eventually leads to cervical cancer. This, however, warrants further investigation.

In conclusion, we have uncovered an intriguing liaison among let-7i-5p, miR-181a-2-3p and EGF/PI3K/SOX2 axis, which is vital for the maintenance of CSCs in cervical cancer (Fig. 6). The further studies on cervical cancer tissue samples and *in vivo* may help develop let-7i-5p, miR-181a-2-3p or SOX2 as potential targets for therapy against cervical CSCs.

## Electronic supplementary material

Supplementary Information
