# Supplementary material for: let-7i-5p, miR-181a-2-3p and EGF/PI3K/SOX2 axis coordinate to maintain cancer stem cell population in cervical cancer
Source: Sci Rep. 2018 May 18;8:7840. doi: 10.1038/s41598-018-26292-w (PMC5959917; doi:10.1038/s41598-018-26292-w)
Supplement: Supplementary file 1 — Supplementary Information [file 41598_2018_26292_MOESM1_ESM.pdf]

let-7i-5p, miR-181a-2-3p and EGF/PI3K/SOX2 axis coordinate to maintain cancer stem cell population in cervical cancer

Ravindresh Chhabra

Department of Biotechnology, Panjab University, Chandigarh, 160014

Address for correspondence:

Dr. Ravindresh Chhabra

Assistant Professor (DST-Inspire faculty)

Department of Biotechnology,

Panjab University, Sector-25

Chandigarh-160014 (India)

E-mail : ravindresh@pu.ac.in
: ravindreshchhabra@gmail.com

Supplementary Table 1: The cDNA primers and the real time PCR primers for miRNAs

miRNA	cDNA primer	Real Time Forward Primer
let-7i-5p	CTCAACTGGTGTCGTGGAGTCGGCAATTCAGTTGAGAACAGCAC	ACACTCCAGCTGGGTGAGGTAGTAGTTTGT
miR-145-5p	CTCAACTGGTGTCGTGGAGTCGGCAATTCAGTTGAGAGGGATTC	ACACTCCAGCTGGGGTCCAGTTTTCCCAGGA
miR-181a-2-3p	CTCAACTGGTGTCGTGGAGTCGGCAATTCAGTTGAGGGTACAGT	ACACTCCAGCTGGGAACATTCAACGCTGTC
miR-615-3p	CTCAACTGGTGTCGTGGAGTCGGCAATTCAGTTGAGAAGAGGGA	ACACTCCAGCTGGGTCCGAGCCTGGGTCTC
miR-663a	CTCAACTGGTGTCGTGGAGTCGGCAATTCAGTTGAGGCGGTCCC	ACACTCCAGCTGGGAGGCGGGGCGCCGCGG
Universal Real Time Reverse Primer	GTGTCGTGGAGTCGGCAATTC	

Supplementary Table 2: The real time PCR primers for mRNAs

Transcript	Real Time Forward Primer	Real Time Reverse Primer
18s rRNA	GTAACCCGTTGAACCCCATT	CCATCCAATCGGTAGTAGCG
ABCA2	AGATGGACAAGATGATCGAG	GCTTGTACTTCAGGATGAGG
ABCG2	CACAAGGAAACACCAATGGCT	ACAGCTCCTTCAGTAAATGCCTTC
c-Myc	GGAACGAGCTAAAACGGAGCT	GGCCTTTTCATTGTTTTCCAACT
CD49f	GAAGGTGGCTGCGGTAGC	GGCTGTGCAAAACAGGAGC
KLF4	ACCAGGCACTACCGTAAACACA	GGTCCGACCTGGAAAATGCT
SOX-2	CGAGTGGAAACTTTTGTCGGA	TGTGCAGCGCTCGCAG

Supplementary File 1: Additional details on the Western Blotting experiment

All antibodies used for this study were purchased from Santa Cruz Biotechnology, USA. The catalog numbers and the lot numbers of the antibodies used are mentioned below:

1. SOX2 (sc-20088, Lot No L1412)
2. PI3k-p55γ (sc-376615, Lot No I1216)
3. Akt1/2/3 (sc-8312, Lot No J2615)
4. p-Akt1/2/3 (sc-514032, Lot No J2815)
5. HMGA2, also known as HMGI-C (sc-130024, Lot No B2812)
6. GAPDH (sc-25778, Lot No I0413)

The blots were developed using the enhanced chemilumiscence ECL western blot detection system from Pierce (Catalog No. 32209, Lot No RJ241236). The blots were then scanned using Azure Biosystems cSeries Image Capture software at default settings. The prestained protein ladder (Thermo Scientific PageRuler, Product No. 26616, Lot No 00204165) was used in all cases for making sure the antibody was capturing the correct sized band in the western blot. For certain blots, the only tool used for enhancing the quality of the blots was rotation and/or brightness/contrast settings for the image in the Microsoft Powerpoint software. Whereever such enhancement was made, it was done on the entire blot and never on any specific sample. For some experiments, the blots were stripped using 2% SDS, 62.5mM TrisCl (pH6.8) and β-mercaptoethanol and then probed for other antibodies. The experiments were repeated multiple times but only one of them is shown in the final figure.

Raw unprocessed original western blot scans shown in the Fig. 2b

1

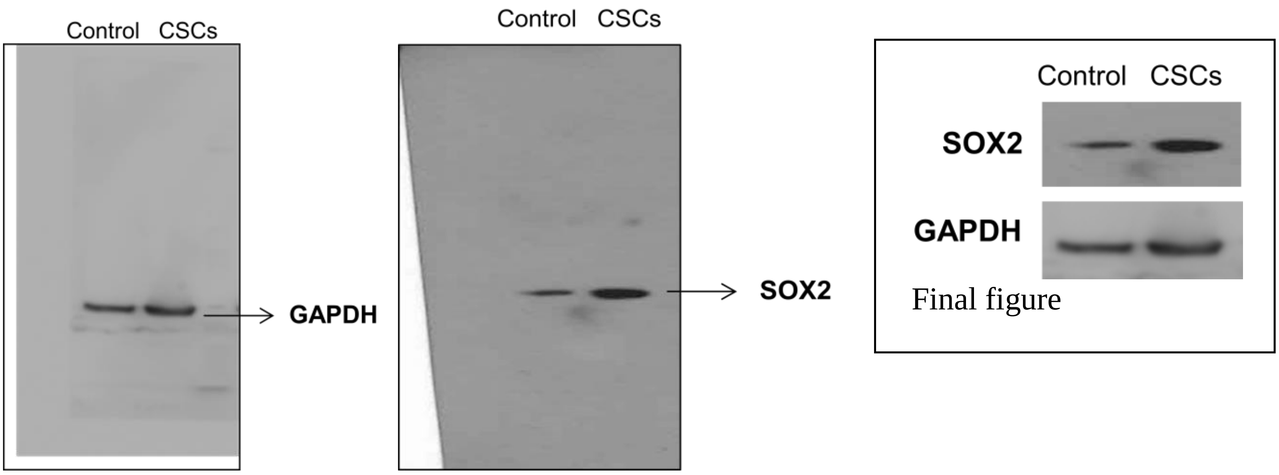

These are intact blots and the band was matched with the molecular weight of the protein using prestained ladder used in the gel

Raw unprocessed original western blot scans shown in the Fig. 5d

2

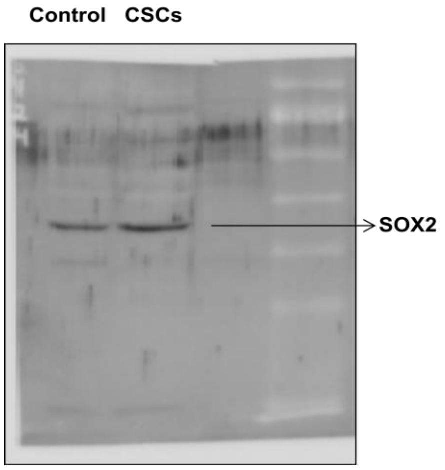

This blot was cropped prior to blotting, to probe for 2 proteins simultaneously

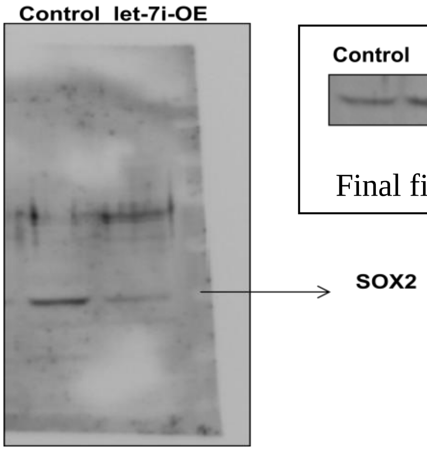

This is intact blot

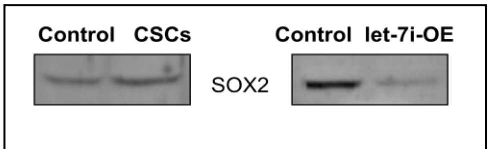

Final figure

3

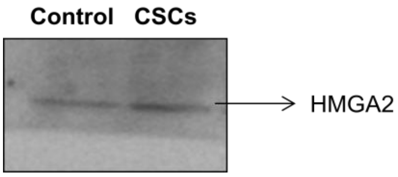

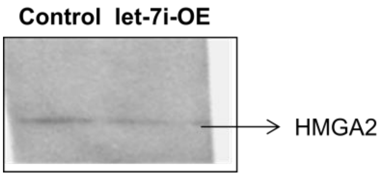

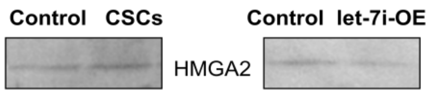

Final figure

Both these blots were cropped prior to blotting, to probe for 2 proteins simultaneously

4

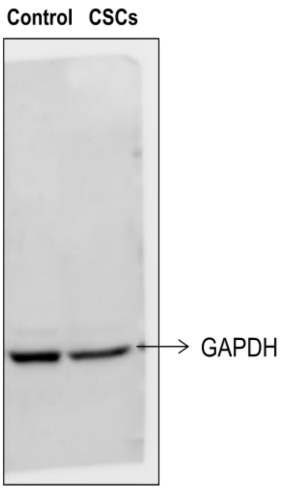

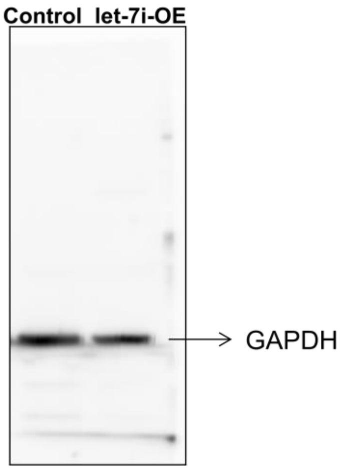

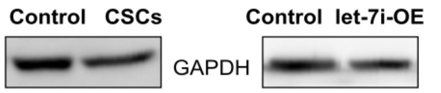

Final figure

The intact blots were used for probing against GAPDH but sometimes they were used after stripping the blots with 2% SDS, 62.5mM TrisCl (pH6.8) and β-mercaptoethanol.

Raw unprocessed original western blot scans shown in the Fig. 5e

5

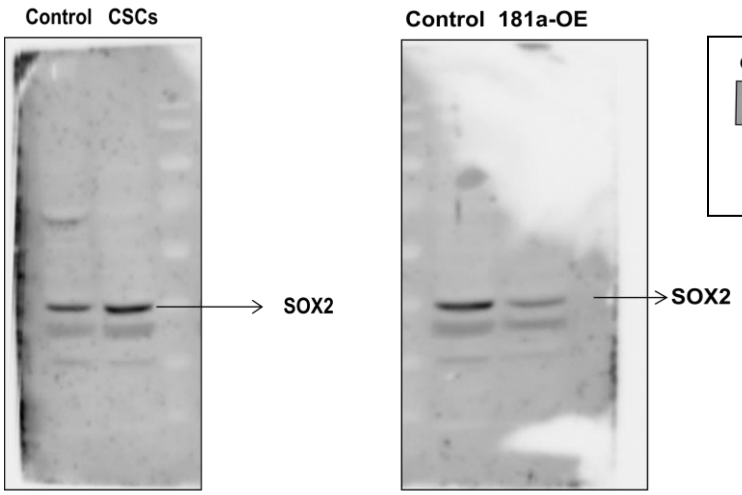

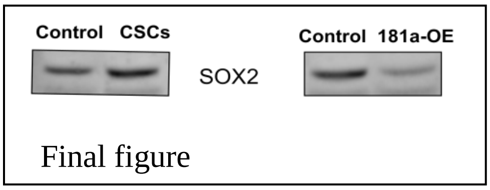

Both of them are intact blots without any cropping

6

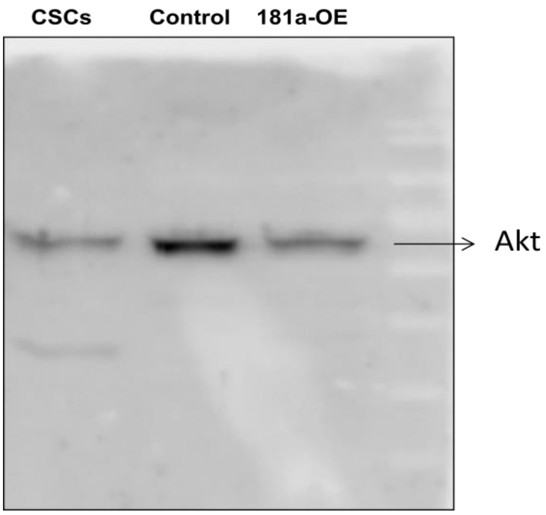

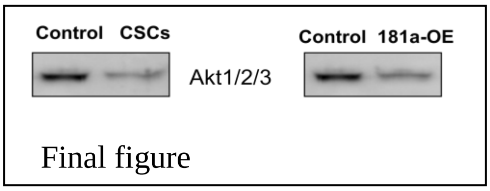

This is intact blot but to represent it in the final figure the same “Control” has been used with “CSCs” and with “181a-OE”

7

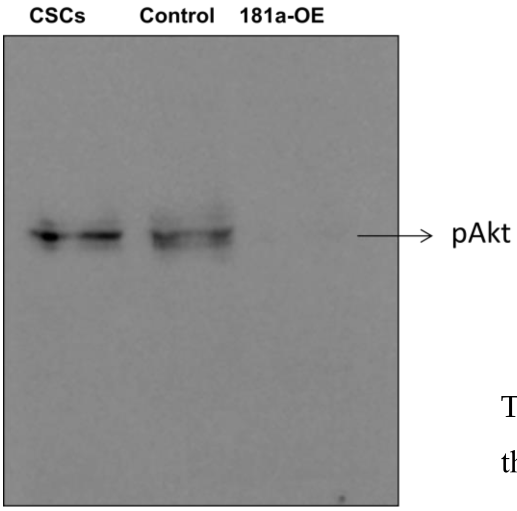

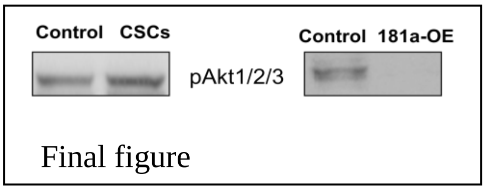

This is intact blot but in the final figure only the “Control” has been used with “181a-OE”

The following blot was used for “Control” with “CSCs”

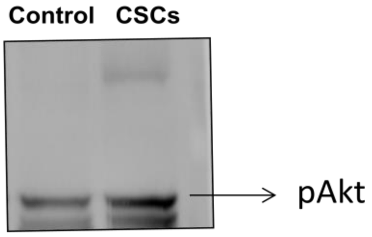

This blot was cropped prior to blotting and is shown in the final figure. An alternative intact blot for pAkt is shown below:

8

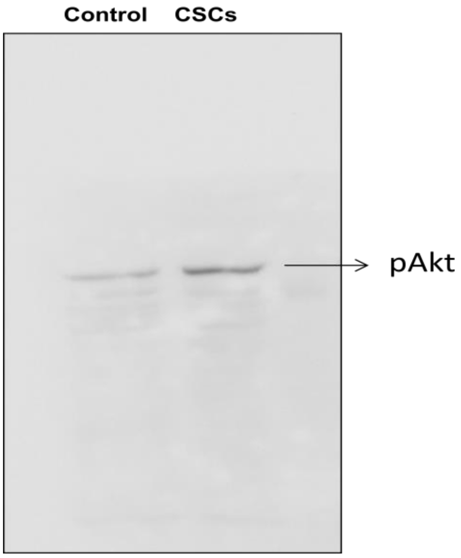

9

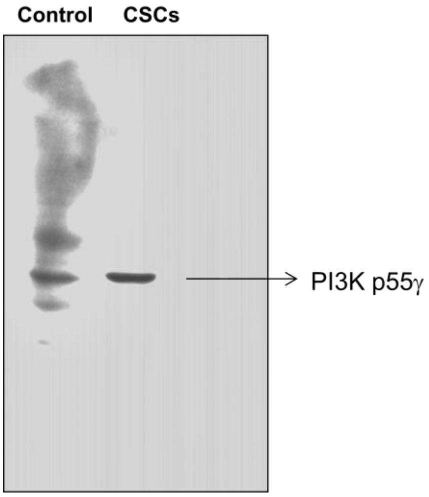

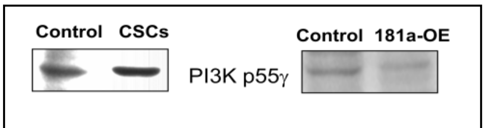

Final figure

This is an intact blot and the band was matched with the molecular weight of the protein using prestained ladder used in the gel

10.

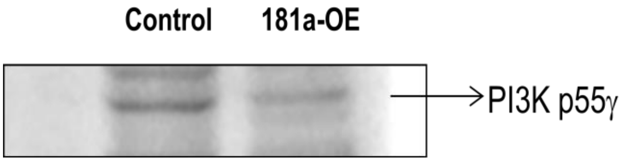

This blot was cropped prior to blotting and is shown in the final figure. An alternative intact blot for PI3Kp55γ is shown below:

11

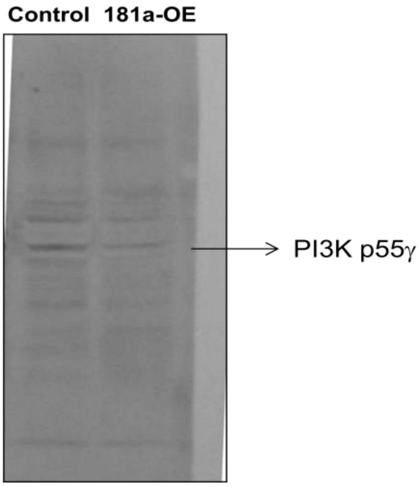

12

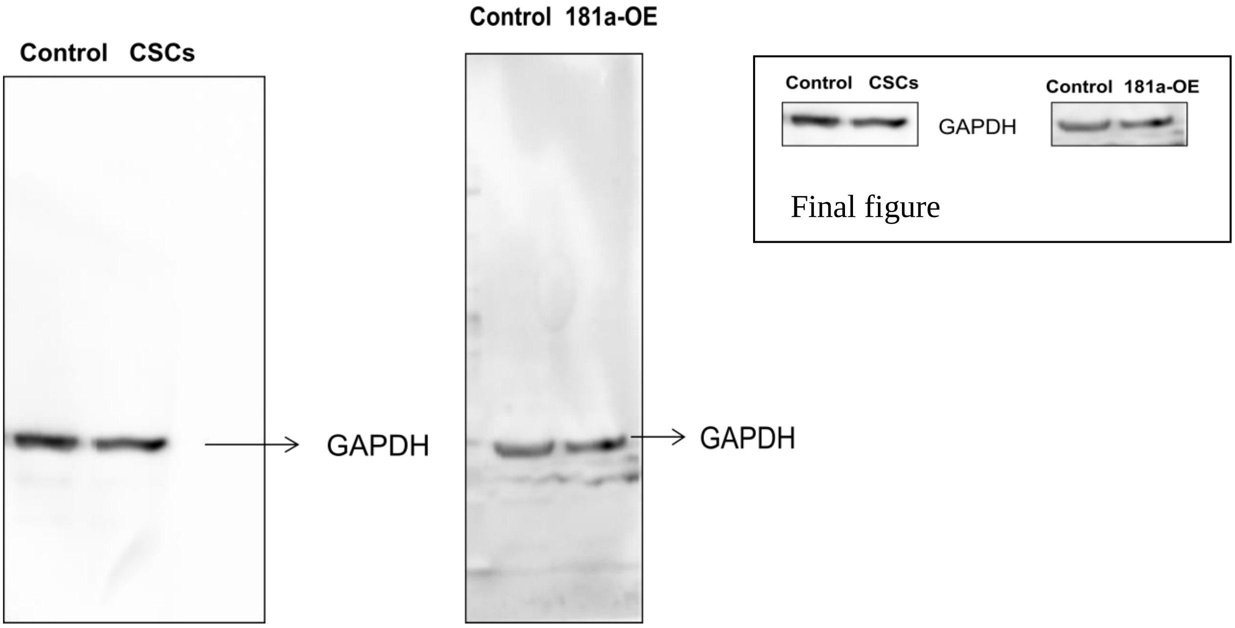

The intact blots were used for probing against GAPDH but sometimes they were used after stripping the blots with 2% SDS, 62.5mM TrisCl (pH6.8) and β-mercaptoethanol.
